# Should describing a new species be science or data release? Rethinking introductions in fungal taxonomy

**DOI:** 10.3897/imafungus.17.186895

**Published:** 2026-06-24

**Authors:** Michaela Caboňová, Sajeewa S. N. Maharachchimbukura, Chance R. Noffsinger, Miroslav Caboň, Xiang-Lin Li, Annemieke Verbeken, Marc Stadler, Chayanard Phukhamsakda, Yasmina Marin-Felix, Laura Guzman-Davalos, Nalin N. Wijayawardene, Slavomír Adamčík, Kevin D. Hyde

**Affiliations:** 1 Laboratory of Molecular Ecology and Mycology, Institute of Botany, Plant Science and Biodiversity Center, Slovak Academy of Sciences, Dúbravská cesta 9, 845 23 Bratislava, Slovakia Centre of Excellence in Fungal Research, Mae Fah Luang University Chiang Rai Thailand; 2 School of Life Science and Technology, Center for Informational Biology, University of Electronic Science and Technology of China, Chengdu 611731, China Institute of Microbiology, Technische Universität Braunschweig Brunswick Germany; 3 Department of Plant Sciences and Plant Pathology, Montana State University, Bozeman, MT 59717, USA College of Biology and Food Engineering, Qujing Normal University Qujing China; 4 Department of Biology, Ghent University, K.L. Ledeganckstraat 35, B-9000 Gent, Belgium Department of Plant Sciences and Plant Pathology, Montana State University Bozeman United States of America; 5 Department Microbial Drugs, Helmholtz Centre for Infection Research, Inhoffenstrasse 7, 38124, Brunswick, Germany Department Microbial Drugs, Helmholtz Centre for Infection Research Brunswick Germany; 6 Institute of Microbiology, Technische Universität Braunschweig, Spielmannstraße 7, 38106, Brunswick, Germany Plant Science and Biodiversity Center, Slovak Academy of Sciences Bratislava Slovakia; 7 Centre of Excellence in Fungal Research, Mae Fah Luang University, Chiang Rai 57100, Thailand Center for Informational Biology, University of Electronic Science and Technology of China Chengdu China; 8 Departamento de Botánica y Zoología, CUCBA, Universidad de Guadalajara, Apdo. postal 1-139, Zapopan, Jal., 45147, Mexico Faculty of Natural Sciences, Comenius University in Bratislava Bratislava Slovakia; 9 Center for Yunnan Plateau Biological Resources Protection and Utilization, College of Biology and Food Engineering, Qujing Normal University, Qujing, Yunnan 655011, China Department of Biology, Ghent University Gent Belgium; 10 Department of Botany, Faculty of Natural Sciences, Comenius University in Bratislava, Révová 39, 81102, Bratislava, Slovakia Departamento de Botánica y Zoología, CUCBA, Universidad de Guadalajara Zapopan Mexico

**Keywords:** Data release, fungal diversity, hypothesis-based research, integrated taxonomy, polyphasic approach, study aims, taxonomic best practices

## Abstract

The Kingdom *Fungi* is estimated to contain several million species and accounts for a large portion of global biodiversity. However, only a small proportion of these fungal species are currently known to science. Ongoing habitat destruction and the resulting loss of diversity due to human activities have created a pressing need to document fungal communities and describe new fungal species. In this study, we analysed publications describing new species from 2020 through 2024 in the basidiomycete genera *Amanita*, *Cortinarius*, and *Russula*, and the ascomycete genera *Aspergillus*, *Fusarium*, and *Pestalotiopsis*. These genera, each containing hundreds or even thousands of known species, rank among the most species-rich groups globally. Our analyses show that in these genera, approximately 60% of 591 new species described in basidiomycetes and 58% of 322 new species of ascomycetes were published in taxonomic papers dealing with a few species in closely related groups (single lineage), but there has been an increasing trend to publish new species in publications containing multiple unrelated new species (cumulative and online release publications). In single lineage publications describing new species, the introduction section often provides only a general description of the genus, omitting important background information for the new or closely related species often mentioning the name of the newly described species to indicate the intent to describe it. However, the formal description and naming should only appear in the taxonomy section as a result of the study. Based on our estimates of the completeness and quality of introductions in single lineage publications, only 25% received scores above 50 points out of a potential 100 points, which is half the expected information needed to assess new species hypotheses. The majority of new species hypotheses are likely based on molecular sequence data, neglecting morphological or ecological components. Due to incomplete background information and poorly articulated aims, many publications resemble data releases rather than hypothesis-driven scientific studies. Based on a thorough review of publications and the authors’ combined experience, we provide recommendations to improve the quality of introductions when describing new species. We outline the importance of complete background information for new species descriptions that link available data with individual study aims. Well-formulated, hypothesis-driven research enhances publication quality, improves clarity for readers, and increases the likelihood of acceptance, while also helping to avoid taxonomic ambiguities and redundant manuscript structure. For studies focused on a single lineage that includes more than five species, we recommend emphasizing lower-rank infrageneric groups (e.g., subgenera, sections, or subsections) in the introduction rather than providing detailed descriptions of individual species. Cumulative and online release publications are efficient tools for documenting and exploring fungal diversity. However, they have limitations, and their long-term contribution to understanding species boundaries will depend on the quality of the data presented and how well they are integrated with more comprehensive, traditional taxonomic studies.

## Introduction

### Significance of new species descriptions

Fungi inhabit every ecosystem on Earth, serving important ecological roles (e.g., saprotrophs, mutualists, and parasites) and providing invaluable benefits to medicine and industry (Hyde et al. 2019). Fungi are highly diverse, and global species richness is estimated to range from 2.2 to 13.2 million (Hyde et al. 2024a), yet most species remain undescribed. Human-driven land-use intensification and habitat destruction highlight the pressing need to document new fungal species, especially since many fungal diversity hotspots remain poorly protected worldwide (Van Nuland et al. 2025). Taxonomists face a critical challenge to identify efficient ways to discover and define species before ongoing environmental degradation results in further biodiversity loss (Costello et al. 2013; Maharachchikumbura et al. 2021; Hyde et al. 2024b; https://www.mycobank.org/Stats%20page). However, comparatively little attention has been paid to how new species are framed, justified, and contextualised within taxonomic publications, particularly in the introduction sections. Without a thorough understanding of fungal diversity, species with critical medicinal, economic, and ecological importance may be lost before formal scientific description (Mapook et al. 2022).

### Standards and good practice

The requirements for effective and valid publication of new species were outlined in the International Code of Nomenclature for algae, fungi, and plants (Turland et al. 2018, 2025). In relation to taxonomy, recommendations for the description of new fungal species are summarised by Aime et al. (2021). Among these, species concepts based on a polyphasic approach (integrated taxonomy) combining phylogenetic (DNA sequence based), phenotypic (including morphology, ecology, phenology, and geographic distribution), and biological (based on mating tests of fungal strains in culture) components, which support species delimitation are highly recommended (Boekhout et al. 2021; Jayawardena et al. 2021; Lücking et al. 2021). Furthermore, fungal taxonomy is regulated by peer-review (i.e., publication quality), journal standards, available funding, and community-specific practices (Loizides et al. 2022). The available data for individual species vary considerably across the kingdom *Fungi*, which represent a very heterogeneous group in terms of evolutionary history and ecology (or their trophic strategy). Therefore, the application of different standards for species descriptions and their global adoption is needed for specific fungal groups (Cao et al. 2021). Despite these widely accepted recommendations, their implementation in everyday taxonomic practice remains inconsistent.

### Problems and consequences

Currently, many new species descriptions rely mainly on molecular data, but the labelling of sequence metadata in public databases is often incorrect or confusing (Hofstetter et al. 2019; De Lange et al. 2023). There is a pressing need for correctly identified and validated publicly available sequences for sequence-based fungal identifications (Kennedy et al. 2022; Abarenkov et al. 2024). Many descriptions of older taxa are based on morphology, lack sequence data and are often poorly characterised, hampering the description of new species that are similar or related (Koukol and Delgado 2021). Older names often require the study of type collections, which can be challenging due to difficulties obtaining loaned specimens, securing permission for destructive sampling, variable collection quality, and limited availability of expertise from specialist mycologists (Dayarathne et al. 2016). While describing a new species from a single collection is generally not considered best practice, formal description is justified when the species is difficult to recollect, and the available material provides robust and clear evidence of a novel taxon (Seifert 2017; Cazabonne et al. 2024). These data-related limitations complicate species recognition, but they alone do not account for the incomplete information provided in many publications of new species.

Despite well-defined requirements for new species descriptions and frequent promotion of good practices, many recent publications describing new species lack important information. For example, in *Botryosphaeriaceae*, 60% of the descriptions are outdated or meet only the minimum requirements for publication and only 50% of authors provide sufficiently accessible and reproducible data to support new species descriptions (Batista et al. 2022). Many publications with new species descriptions lack connections to broader ecological or evolutionary questions, limiting their contribution to our understanding of biodiversity. In addition, many introductions to new species descriptions restate that the new species is being described and summarise the methods used, content that often duplicates information in other sections of the manuscript. As a result, many introductions read as summaries of results rather than as frameworks for hypothesis testing and consequently resemble data releases more than hypothesis-driven scientific studies.

Against this background, this study aims to evaluate how new species hypotheses are framed and supported in taxonomic publications, with particular emphasis on the completeness and structure of introduction sections. We analysed publications introducing new fungal species in six well-known and species-rich genera of basidiomycetes and ascomycetes published from 2020 through 2024. We discuss the types of information that should be included in an introduction and provide recommendations for constructing well-developed introductions in taxonomic papers, emphasising the importance of meaningful background context, strong justifications, explicit aims, and testable hypotheses when describing new species.

## Materials and methods

### Sorting new species descriptions

To assess the quality and completeness of introductions in publications with new species descriptions, we selected three ectomycorrhizal genera of basidiomycetes: *Amanita*, *Cortinarius* (*sensu lato*), and *Russula*, and three non-mutualistic genera of ascomycetes: *Aspergillus*, *Fusarium*, and *Pestalotiopsis*. Ectomycorrhizal basidiomycetes form mutualistic associations with plants and occur across diverse soil ecosystems almost worldwide (Tedersoo et al. 2022). *Cortinariussensu lato* and *Russula* are among the most diverse ectomycorrhizal genera globally, and *Amanita* is also known to be relatively diverse; they all inhabit similar ecosystem types and associate primarily with woody plant partners. The selected filamentous ascomycetes are economically important fungal pathogens, while some *Aspergillus* species weaken the human immune system and cause allergies (Visagie et al. 2024). Species of *Fusarium* also produce mycotoxins harmful to animals and are found as contaminants in human food (Sun et al. 2020; Crous et al. 2021). Species of *Pestalotiopsis* cause diseases of various crops and produce a plethora of secondary metabolites, some of which are important for bioremediation and anti-tumour activities (Zhang et al. 2025). These genera were selected to enable comparisons across contrasting ecological strategies, taxonomic practices, and publication cultures within contemporary fungal systematics.

New species published from 2020 through 2024 for each of the selected fungal genera were obtained by searching MycoBank (https://www.mycobank.org/) (Suppl. material 1). Only validly published names of species new to science were included. Names published that were below species rank, invalid, or assigned as taxonomic synonyms of older, validly published names were not included. Species names originally published under the six focal genera of this study, but later combined elsewhere, were included. If the authors validated invalid names, these species were included and linked to their earlier descriptions. For *Cortinarius*, we also included the genera *Aureonarius*, *Austrocortinarius*, *Calonarius*, *Cystinarius*, *Hygronarius*, *Mystinarius*, *Phlegmacium*, *Thaxterogaster*, and *Volvanarius*, all of which were historically classified within *Cortinariussensu lato* prior to recent taxonomic studies (Liimatainen et al. 2022; Gallone et al. 2024). These taxa were included to account for inconsistent use of updated classifications across the literature and databases.

Publications were sorted into four categories: A) species descriptions without introductions or further comments (e.g., the online publications at Index Fungorum), B) cumulative publications with a broad range of unrelated species descriptions (e.g., Fungal diversity notes, Fungal planet description sheets, Index of Australian Fungi, Mycosphere notes, The genera of fungi), C) single lineage publication, which are a traditional type of taxonomic publications usually dealing with a few species or a monophyletic group within a genus, and D) monographs (books, book chapters, or comprehensive articles that included all known species from a particular geographical area or substrate) addressing higher-rank taxonomic groups, such as genera or families. Further analysis focused on single lineage publications because the introductions of these studies typically formulate hypotheses and research objectives within the context of a single taxonomic group.

### The quality and completeness of introductions in single lineage publications

We sorted single lineage publications (category C) based on whether the introduction listed new species names or mentioned that new species are described without providing their names. This was done because listing the names of new species in the introduction does not align with hypothesis-based research.

In single lineage publications, the quality and completeness of introductions were evaluated based on whether they included information about the focal taxonomic group, described the available data, and effectively linked these components to the aims of the study and new species hypotheses. We scored the presence of this information across five categories (outlined in Box 1), each with a maximum of 20 points, for a total possible score of 100. Each category was scored based on the presence and completeness of the information provided in the introduction as follows: 0 for missing information, 5 for a weak explanation, 10 for intermediate completeness, not explaining the topic background, 15 for almost complete background, and 20 for a complete and well-presented background for the category.

**Box 1.** Categories and criteria for estimating the completeness of introductions in new species publications. These evaluated categories represent preliminary observations and published data used for the explanation of the new species hypothesis.

1. Phylogenetic placement: Placement of species within the genus, or clear characterisation of the group of interest.
Are the focal taxa clearly placed within the genus, preferably at the lowest relevant infrageneric rank (e.g., subgenus, section, subsection, species complex), using phylogenetic and/or morphological evidence?
2. Closely related species: Review of species with close phylogenetic relationships in published studies that include the group in question.
Are relevant phylogenetic studies adequately summarised?
3. Morphology: Differentiation of morphological characters between closely related or similar species within the group.
Are individual morphological characters used for species delimitation within the group discussed?
4. Ecology and biogeography: Ecology and distribution of new species compared to closely related species within the group.
Is there evidence from previous studies suggesting the possibility of ecological speciation or geographic isolation? Does the introduction provide relevant ecological or biogeographic information that may support species delimitation (e.g., host specificity, habitat preference, geographic isolation)?
5. Hypothesis: Explanation of species hypothesis and study aims.
Is the species hypothesis explicitly defined, identifying the preliminary diagnostic characters (morphological, genetic, or ecological) that may distinguish this lineage from known relatives? Are the research aims clearly articulated, and do they provide a logical framework for interpreting the observed biological variation? Does the hypothesis address the mechanisms of divergence (e.g., geographic isolation, niche partitioning, reproductive barriers) that led to the formation of the new species?

## Results

### Sorting publications with new species descriptions

**Figure 1.**
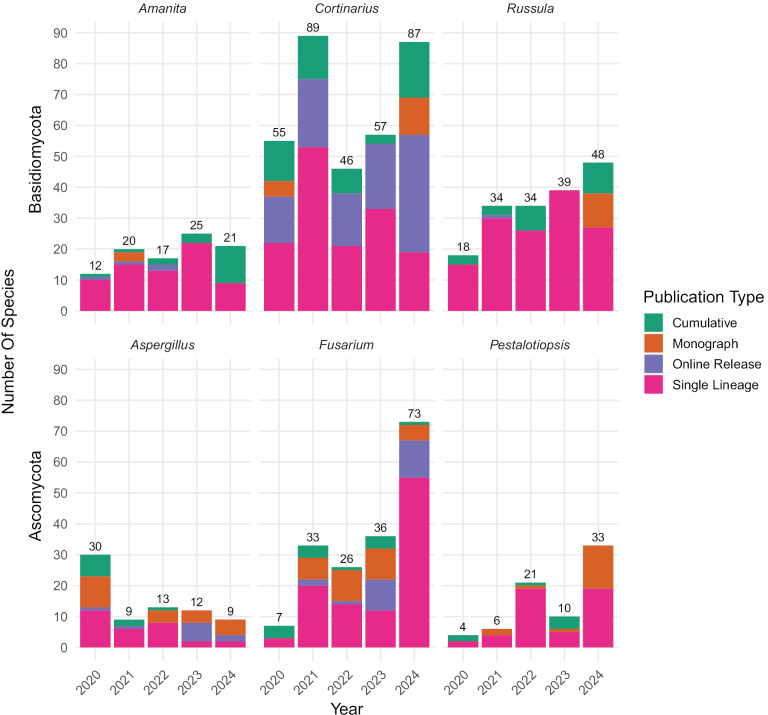
New species of selected basidiomycete and ascomycete genera described from 2020 through 2024 and sorted in four publication categories.

Between 2020 and 2024, a total of 591 new species of *Amanita*, *Cortinarius*, and *Russula* were described across 219 publications (Table 1, Fig. 1). Of these, 117 species were published through online release, 88 in cumulative publications, 355 in single lineage publications, and 31 in monographs. Among the 219 publications, the majority (167) focused on a single lineage. This confirms that single lineage publications remain the dominant format for introducing new fungal species. In the same period, a total of 322 new species of *Aspergillus*, *Fusarium*, and *Pestalotiopsis* were described across 138 publications (Table 1, Fig. 1). Of these, 35 species were published through online release, 31 in cumulative publications, 183 in single lineage publications, and 73 in monographs. Among the 138 publications, approximately half (67) focused on a single lineage, while the remainder were distributed nearly evenly across the other three publication categories (23 cumulative, 24 online release, 21 monographic). In our dataset, we also encountered new species that were later recognised as taxonomic synonyms of older names. Some were published in data releases or cumulative publications, while others were described in single lineage publications with poor quality introductions and were not included in further analysis.

**Table 1.** New species of selected basidiomycete and ascomycete genera described between 2020 and 2024. The number of new species for each genus is sorted into four publication categories.

Publication type	* Amanita *	* Cortinarius *	* Russula *	* Fusarium *	* Aspergillus *	* Pestalotiopsis *	Total
Online Release	4	112	1	25	10	0	152
Cumulative	18	50	20	14	10	7	119
Single Lineage	68	150	137	104	30	49	538
Monographs	3	17	11	32	23	18	104
Total	93	329	169	175	73	74	913

### Quality and completeness of introductions in single lineage publications

**Figure 2.**
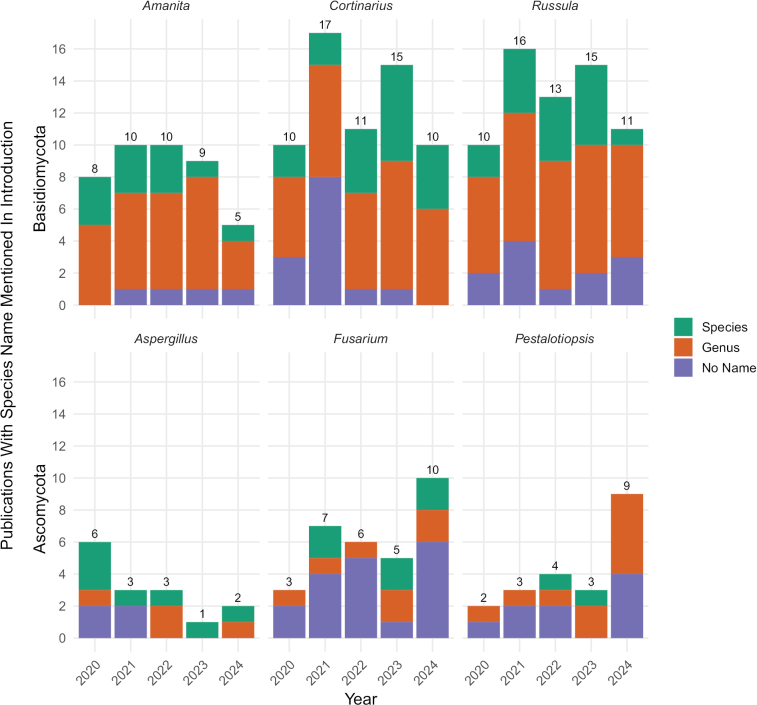
Single lineage publications (category C explained in Materials and methods) published in 2020–2024 for the assessed basidiomycete and ascomycete genera sorted by the mention of a newly described species in the introduction. In the legend: Species indicates that the newly described species is/are mentioned in the introduction; Genus indicates that the introduction mentioned a new species is being described, but did not mention the species epithet; No name indicates no mention of the new species or that a species is being described in the introduction.

Many single lineage publications did not clearly state the primary justification for describing new species. Specifically, 134 basidiomycetes studies (80%) and 38 ascomycetes studies (57%) failed to articulate the underlying species hypothesis (whether based on molecular, morphological, ecological, or geographical lines of evidence) and instead focused primarily on presenting descriptive information rather than explicit study aims. Furthermore, 40 basidiomycetes publications (24%) and 15 ascomycetes publications (22%) listed the name of a new species directly within the introduction (Table 2, Fig. 2). Unlike basidiomycetes, new species of ascomycetes were often included in large articles based on systematic and extensive material collections from defined substrates or geographic regions. The ascomycetes described in these large publications were assigned to higher taxonomic groups, such as genera or families, with little emphasis on infrageneric classifications. Most comprehensive studies organised by substrate or geography were treated as monographs. However, comprehensive studies on multiple species classified within a strictly defined infrageneric group were treated as single lineage publications.

**Table 2.** Single lineage publications (category C explained in Materials and methods) sorted by mention of new species in introductions (MNSI).

MNSI	* Amanita *	* Cortinarius *	* Russula *	* Aspergillus *	* Pestalotiopsis *	* Fusarium *	Total
Species	9	16	15	6	7	2	55
Genus	26	31	37	8	4	11	117
No Name	5	16	12	17	4	8	62

**Figure 3.**
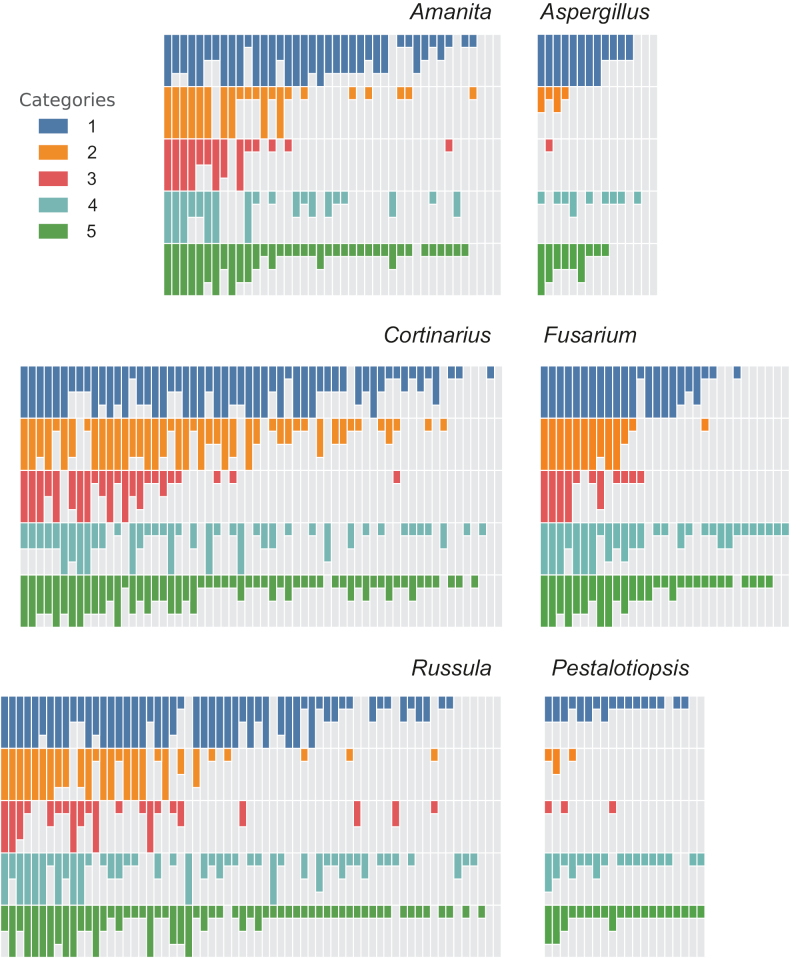
Evaluation of introduction completeness based on the five categories assessed according to criteria in Box 1. Categories: 1. Phylogenetic placement, 2. Closely related species, 3. Morphology, 4. Ecology and biogeography, and 5. Hypothesis. Scoring of individual publications is explained in Suppl. material 2.

**Figure 4.**
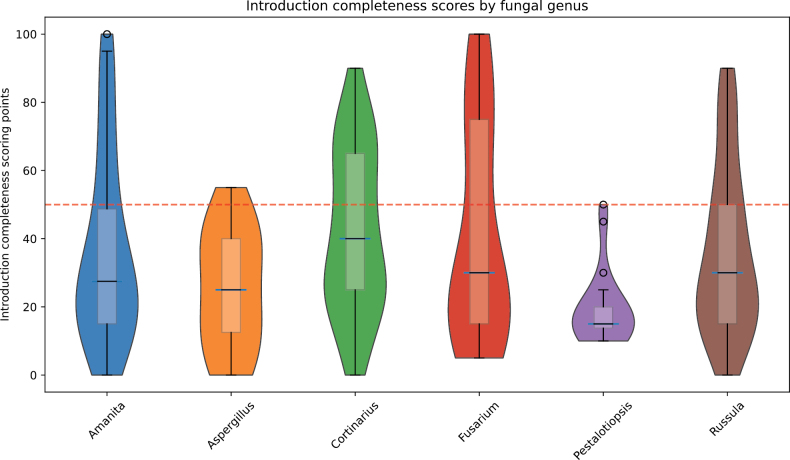
Introduction completeness in publications with new species descriptions based on scoring criteria outlined in Box 1. The red dotted line indicates a score of 50 and represents a relatively complete introduction section.

Introductions for single lineage publications often started with a general description of the genus in question, which often represented a large portion of the introduction. Among the five evaluated categories (Box 1) for all genera, the phylogenetic placement of new species was the most complete. The category morphology was the least complete (Fig. 3, Suppl. material 2). In general, we considered introductions with scores above 50 to be relatively complete, but 75% of the publications scored below this threshold (Fig. 4).

### Phylogenetic placement within the genus and closely related species

The taxa assessed were often not assigned to lower-rank infrageneric groups. This was particularly common among the basidiomycetes evaluated, especially in publications that included new descriptions of multiple, phylogenetically unrelated species within the same genus. The classification within the genus was commonly based on molecular analyses and less often on morphological criteria. When lower-rank infrageneric classifications were included, the authors rarely specified whether they were based on morphology or phylogeny. Additionally, in some cases, it was difficult to determine if species were placed in the lowest-rank group possible within the genus. In some cases, the target group was defined by the geographic origin of the samples or their plant partners, rather than by taxonomic classifications. In ascomycetes, the absence of individual species classifications within the genus was more frequent compared to the basidiomycetes assessed, likely because the sampling and target groups were defined by substrate or geographic area and included multiple species from a predefined group.

The known diversity within a target group was not always specified. Estimating diversity remains difficult for many highly diverse groups because numerous older species lack sufficient data (e.g., DNA barcodes or comprehensive morphological descriptions), hindering accurate assessments of current species richness. When species diversity was considered, it was typically addressed at a global scale and often lacked data relevant to the study area or substrate.

### Morphology

It is likely that many introductions used a sequence-based species hypothesis, and neglected morphology. Morphological character descriptions were usually limited to general descriptions above species rank (e.g. subgenera or sections) and rarely used for defining differences among closely related species.

### Ecology and biogeography

Introductions frequently included general descriptions of the geographic area under study, but often did not link this information to descriptions of new species. The ecological and biogeographic information was largely descriptive and lacked hypothesis-driven analyses, such as assessments of species endemism or host-plant specificity. Biogeographic assessment was often based on the countries of sample origin, but these designations do not always align with species-specific biogeographic patterns. Some countries span multiple biogeographic regions due to diverse habitats, while others encompass only a single or partial region. For example, the diversity in the southeastern Himalaya is similar to that in parts of China, India, Nepal, and Pakistan, and the diversity in southeastern subtropical China (Yunnan) is similar to that in Japan, Thailand, and other countries in Southeast Asia. Some studies defined larger geographic areas that were heterogeneous in climate, host range, and/or soil conditions, thus providing no specific information for the studied material or putative new species.

### Hypothesis and aims

Most publications used general, imprecise arguments to justify descriptions of new species, often citing morphology and phylogeny as the basis for defining new species. The species hypothesis (the primary reasons why new species are expected) was usually not explained but was often apparent from the context of the introduction. Aims were not always defined, and in many cases, the stated goal was to describe a new species. The last sentences of the introduction often reiterated that new species are being described (sometimes providing their names), duplicating information already presented in the results.

## Discussion

Introductions for scientific papers should provide the background required to understand the purpose of a study. Scientific publications differ from data release by having clearly defined hypotheses and aims (Penev et al. 2017). It is essential to link data generated within the study with the study’s aims. Readers expect to get an overview of current knowledge on the topic. In particular, introductions for taxonomic publications should provide a clear characterisation of the group of interest, a review of previously published studies, and some morphological or molecular characteristics that clearly justify the description of a new species. Authors should explain the evidence supporting its recognition and articulate the rationale guiding the study from initial discovery to formal description. Publications with new species descriptions should include information on why the species is thought to be novel, but also involve multiple approaches. Traditional species descriptions have focused on morphological traits, ecological context, and sometimes chemical reactions that distinguish one species from another, while prevailing arguments in recent publications are sequence-based.

In modern fungal systematics, species concepts are based on testable hypotheses generated from available data (Steenkamp et al. 2018; Taylor et al. 2000; Chethana et al. 2021; Cai et al. 2011; Jayawardena et al. 2021). In modern evolutionary biology and systematics, a species name is treated as a hypothesis (i.e., this organism may represent a lineage distinct from related ones). This claim should be supported by available data, including any combination of phylogenetic, morphological, geographical, ecological, or reproductive differentiation (Stengel et al. 2022). Modern taxonomy increasingly takes an integrative approach, combining multiple lines of evidence (including all the above-mentioned components), which is currently considered the gold standard (Boekhout et al. 2021; Jayawardena et al. 2021; Lücking et al. 2021). However, our analysis revealed that many studies likely used a simple sequence-based hypothesis. This is supported by the authors’ relatively well-communicated phylogenetic placement of studied species, and reference to relevant phylogenetic studies (Fig. 4). Other components that provide additional evidence to support species delimitations (morphology, biogeography, ecology) are often insufficiently represented and not directly linked to the project aims.

Species hypotheses based on multiple data sources (sequence, morphology, and ecology) can help prevent the publication of new names for taxa that have already been described (junior synonyms); e.g., morphology-based hypothesis should encourage authors to assess studies on types of closely related taxa (Dayarathne et al. 2016; Koukol and Delgado 2021). For example, some species names in our dataset that are now recognised as junior synonyms were originally published in data releases or cumulative publications, whereas others were described in publications with limited or low-quality introductions. In larger studies that included complex revisions of the target group and multiple new species, explaining the species hypothesis for each species is impractical and might confuse the reader. In such cases, we recommend including detailed descriptions (i.e., phylogenetic placement and relationships, morphology, ecology, geographic distribution, and closely related species) for lower-rank (infrageneric) groups or species complexes in the introduction. See Costa et al. (2024) or Sandoval-Denis et al. (2025) for good examples of detailed infrageneric descriptions in manuscripts that describe multiple new species. In rare cases, we observed explicit hypotheses for each individual species described (e.g., Xie et al. 2023), although the species were not closely related. We recommend this approach when manuscripts describe a relatively small number of species (e.g., 5 or fewer).

Although introductions often omitted detailed phenotypic descriptions and tended to be brief, many also included redundant information. Many introductions included general, generic, or higher-rank information that is well known to mycological readers and can be explained in one or two sentences with proper referencing to previous publications, books, or online resources. For example, Cui et al. (2018) could be cited for *Amanita*, Samson and Varga (2009) for *Aspergillus*, Liimatainen et al. (2022) for *Cortinarius*, Crous et al. (2021) for *Fusarium*, Maharachchikumbura et al. (2014) for *Pestalotiopsis*, and Looney et al. (2018) for *Russula*. The introductions in the studies often contained redundant information already present in the results or conclusion. Based on our assessment, introductions usually lacked a clear description of the molecular and morphological evidence used to describe a new species, study aims, and testable hypotheses. We recommend that authors avoid mentioning the names or numbers of new species being described in the introduction, as this practice does not support hypothesis-based science. Instead, authors should frame their aims around explicit hypothesis testing; for example, assessing whether geographic origin corresponds with variation in DNA sequences and/or ecological traits (Box 2). However, understanding the rationale for testing species hypotheses requires knowledge of which taxa are most closely related or morphologically similar, as well as the geographic and ecological range (or origin) of both the proposed new species and its close relatives, which is why this information needs to be included in the introduction.

**Box 2.** Recommendation for the introduction of single lineage studies (category C in Materials and methods) with new species descriptions.

Mandatory requirements:
• General procedures. Do not state the names of any new species in the introduction. State the names in the abstract and state the number of new taxa described in the paper in the keywords (Schoch et al. 2017). The term “sp. nov.” should be used exclusively at the place where the action takes place, preventing confusion in citation of the exact place of effective publication of new species name (Recommendation 32A.1 of the Code, Turland et al. 2025).
• Duplicity and redundancy. Avoid stating results in the introduction. Any information about new species identity and diagnostic characters should be in the results, taxonomy, conclusions, and abstract sections of the study.
Strongly recommended items:
• Higher rank groups description. In studies submitted to mycological journals, avoid detailed descriptions and discussions of large and well-known fungal genera or families and rather cite reliable sources summarising current global knowledge of the genus or family. However, if the classification has or is rapidly changing, this should be discussed.
• Classification. In genera with infrageneric groups (i.e., subgenera, sections, subsections, species complexes) define these groups (with evidence) for each new species included in the study at the lowest taxonomic rank possible. This can be difficult for some genera, and statements of uncertainty should be included when necessary. Authors should briefly state which gene regions (e.g., ITS, LSU, *rpb2*) support the placement and mention the key morphological traits that match that group. Phylogenetic or taxonomic studies that support this classification, preferably the ones that introduced it, should be cited.
• Closest relatives. Specify which species are closely related based on preliminary sequence similarity searches, prioritising type-derived or otherwise validated sequence identifications, and indicate when such comparisons are not possible. Cite phylogenetic publications that include species of the target group. If there is no reliable phylogenetic study for the lineage, clearly explain that the current study is the first to infer close relationships within the group.
• Morphology. Key morphological characteristics of the group of interest should be clearly outlined. Optionally, mention morphological similarities or differences from the closest relatives (based on available sequence data) and the most similar taxa. Indicate whether the species is pleomorphic, meaning it exhibits morphological variation across its life cycle or if varies in response to environmental conditions. Include a statement that assesses whether these traits align with the current practices in the group of interest.
• Hypothesis and aims. Ensure that the species hypothesis (the primary reason to consider the new species description) is clear from the information provided in the introduction. Do not use general statement (e.g., morphology and phylogeny were used to support the new species delimitation) to explain the support for the new species. Formulate aims using specific information about sequence similarity, morphology, biogeography, and ecology of the new species (see examples in Box 3).
Optional points:
• Ecology and biogeography. If possible, explain whether the new species shows host-specificity, habitat preferences, or geographic isolation compared to its close relatives.
• Classification accuracy. Authors should briefly state which gene regions (e.g., ITS, LSU, *rpb2*) support the placement in a section or species complex. In addition to GenBank, it is recommended to search other publicly available sources that contain unique data (e.g., UNITE, GlobalFungi, iNaturalist, MycoMap).
• Integrated (multiphase) approach. Sequence similarity should be interpreted together with morphological, biological (culture-based), ecological, or biogeographic data to support the initial species hypothesis.
• Large single lineage studies. Studies describing multiple (more than five) new species in the same paper should provide a brief summary of the overall diversity they address, group each species clearly according to infrageneric classification, and avoid long lists of species’ names in the introduction (e.g., Costa et al. 2024; Dima et al. 2021).

In this study, we demonstrated relatively low completeness and quality of introductions. Based on our assessment, only one quarter of publications scored above 50 points, which we considered the threshold for good-quality introductions with clearly communicated hypotheses and aims. Clear formulations of hypotheses and aims will link available data to species descriptions, providing a hypothesis-based scientific approach rather than merely releasing the data. Therefore, we provide recommendations to help improve the quality of publications describing new species and their underlying hypotheses (Box 1).

**Box 3.** Examples of new species hypotheses supported by different argumentations. The text is adopted from single lineage publications assessed in this study (Suppl. material 1). To clarify the hypotheses and their context in the following studies, we added or modified text, indicated within brackets.

General examples of components supporting the species hypothesis
• Sequence divergence from the closest relative is below/above a known threshold.
• Key morphological traits differ consistently from the sister clade.
• Collections from a geographically isolated area form a separate, supported clade.
Examples from the literature
• *Amanita* – Davison et al. (2021): “The ITS region does not appear as useful among Australian species. For example, Davison et al. (2017a) found that ITS sequences fail to separate three species from section *Phalloideae*, which differ in spore shape and are geographically separated, and within *A. peltigera*, the base pair divergence among haplotypes from the same individual is between 0.2 and 5.8% (Davison et al. 2020). In this paper, we determine the difference between ITS clones from both secotioid and agaricoid members of section *Arenariae*, to determine whether they show divergence similar to that observed in *A. peltigera*.”
• *Aspergillus* – Huang et al. (2020): “During an investigation of the diversity of alkali-tolerant or alkalophilic fungi and assessment of their enzyme activity in China, two alkali-tolerant isolates of *Aspergillus* were isolated. The phylogenetic analysis based on the combination of BenA, CaM, and ITS sequences suggests an affiliation to *Aspergillus* section *Terrei*.”
• *Cortinarius* – Lam et al. (2024): “During our ongoing studies of Chilean *Cortinarius* species in *Nothofagus dombeyi* forest along the Cordillera de Nahuelbuta, we repeatedly observed a species phenotypically very similar in size and colour to the species *Cortinarius ignotus* Horak from New Zealand, but distinctively characterized by a conspicuous violet-black colour reaction with KOH.”
• *Fusarium* – Lynn et al. (2020): “The taxonomic revision of the *F. fornicatus* complex has led to a situation where the identity of the most common ambrosia beetle infesting *Acacia* spp. in Riau, Indonesia is not known. Likewise, the identity of its fungal symbiont(s) has not been determined.”
• *Pestalotiopsis* – Wang et al. (2024): “During a survey of diseases in Chinese yew, moderate-to-severe incidences of needle spot and stem canker diseases were observed in some planting areas in Guangxi Province in 2020, and several Pestalotioid fungi were isolated from the diseased Chinese yew.” [The preliminary results indicated that the diversity of Pestalotioid species associated with Chinese yew was greater than previously determined.]
• *Russula* - Buyck et al. (2023): [There remains the possibility that *Russula prolifica* described from plantations of *Eucalyptus rubusta* in Madagascar is recently imported to Madagascar with the tree, because this species has never been observed in native habitats of the country.] The only potential Australian candidate of a *Russula* that appears extremely similar in the field to *R. prolifica* was a species presented under the name ‘*R. aff. cyanoxantha*’ in Bougher and Syme (1998) but also referred to there as ‘Multicoloured *Russula*’ and ‘*R. multicolor*’ [nom. inval.].

The online release or cumulative studies formats are simple and can be beneficial for mycologists who are not specialists in the focal group (or want to publish an opportunistic discovery). Even descriptions based on minimal data can provide a valuable foundation for future work (Aime et al. 2021). Nevertheless, as the number of opportunistic single-species descriptions grows, often lacking critical comparison with related taxa, it becomes increasingly important to conduct broader, integrative taxonomic studies. Such studies should incorporate multiple species within the group of interest, ideally represented by samples from across their geographic range (Adamčíková et al. 2025). Therefore, we recommend against the use of online releases or cumulative studies in favour of single lineage or more comprehensive works that contain higher-quality data and systematically improve on our understanding of fungal diversity.

## Conclusions

Introductions in scientific publications are essential for synthesising existing knowledge, clearly stating research aims, and framing tested hypothesis. Our assessment of standard taxonomic studies describing new species found that many introductions lack this hypothesis-driven structure, resulting in puzzling context and limited support for the taxonomic decisions presented. Improving the quality and completeness of introductions is therefore critical for strengthening species delimitation, increasing transparency, and enhancing the scientific rigor of taxonomic work.

While the pace of species discovery in mycology remains a concern, particularly in the face of accelerating biodiversity loss, our results emphasise that quality should not be sacrificed for speed. Well-developed, hypothesis-driven introductions encourage the integration of multiple lines of evidence, reducing the risk of taxonomic errors and poorly supported descriptions. We believe that improving the quality of introductions will increase publication acceptance rates and facilitate further study of the groups in question. In turn, higher-quality foundational studies provide a more robust framework for subsequent research, facilitating greater, successive efforts that more effectively advance our understanding of fungal diversity.

We recommend that authors provide clear background context built on the current literature, integrate multiple data sources where possible, and situate new taxa within broader ecological and evolutionary frameworks, explicitly justify the need for describing new species, and state testable hypotheses and well-defined aims. By prioritising well-structured introductions, taxonomic studies can both improve individual species descriptions and contribute more effectively to the broader synthesis of fungal biodiversity. Although approaches such as rapid or cumulative taxonomy can accelerate species discovery, their long-term value depends on the clarity, rigor, and contextua­lisation established at the onset. Additionally, these publications often suffer from limited or poor review quality because they include heterogenous species from multiple phylogenetically unrelated groups, making it difficult to find reviewers with expertise across all taxa involved.

In this study, we conducted an in-deep review of taxonomic publications with the aim of promoting the implementation of hypothesis-driven introductions in new species descriptions. In the near future, it would be valuable to develop recommendations for the construction and analysis of molecular, morphological, and other data related to species delimitation. However, improving fungal taxonomy is not a simple task. Many new species descriptions appear to be written primarily for mycological audiences, often overlooking broader contexts such as fungal biogeography, ecological function, and biochemistry that would engage the broader scientific community (Durkin et al. 2020). In larger monographic or single lineage studies, integrating biological traits and ecological information into studies would elevate the visibility of mycology within the broader biological sciences and, in turn, help attract greater funding and opportunities for mycological research (Zhou and May 2023).

## Additional information

### Artificial Intelligence (AI) use

The authors accept full responsibility for the content of the manuscript, including the disclosure of any use of AI.

No AI tools were used in the preparation of this manuscript.

### Adherence to national and international regulations

All the fungal strains used in this study have been legally obtained, respecting the Convention on Biological Diversity (Rio Convention).

### Author ORCIDs

M. Caboňová https://orcid.org/0000-0002-2207-6183

S.S.N. Maharachchimbukura https://orcid.org/0000-0001-9127-0783

C.R. Noffsinger https://orcid.org/0009-0003-9658-4572

M. Caboň https://orcid.org/0000-0002-2255-3816

X.-L. Li https://orcid.org/0009-0007-4364-6994

A. Verbeken https://orcid.org/0000-0002-6266-3091

M. Stadler https://orcid.org/0000-0002-7284-8671

C. Phukhamsakda https://orcid.org/0000-0002-1033-937X

Y. Marin-Felix https://orcid.org/0000-0001-8045-4798

L. Guzmán-Dávalos https://orcid.org/0000-0002-4390-3678

N.N. Wijayawardene https://orcid.org/0000-0003-0522-5498

S. Adamčík https://orcid.org/0000-0003-2156-5767

K.D. Hyde https://orcid.org/0000-0002-2191-0762

### Data availability

All of the data that support the findings of this study are available in the main text or Supplementary Information.

## Supplementary materials

Supplementary material 1 Literature sources Data type xlsx Explanation note List of all analysed new species of *Amanita*, *Aspergillus*, *Cortinarius*, *Fusarium*, *Pestalotiopsis* and *Russula* published in 2020-2024 with references. https://binary.pensoft.net/file/1695004This dataset is made available under the Open Database License (http://opendatacommons.org/licenses/odbl/1.0/). The Open Database License (ODbL) is a license agreement intended to allow users to freely share, modify, and use this Dataset while maintaining this same freedom for others, provided that the original source and author(s) are credited. Michaela Caboňová, Sajeewa S. N. Maharachchimbukura, Chance R. Noffsinger, Miroslav Caboň, Xiang-Lin Li, Annemieke Verbeken, Marc Stadler, Chayanard Phukhamsakda, Yasmina Marin-Felix, Laura Guzmán-Dávalos, Nalin N. Wijayawardene, Slavomír Adamčík, Kevin D. Hyde

Supplementary material 2 Points in categories Data type xlsx Explanation note Scoring of publications with new species of Amanita, Aspergillus, Cortinarius, Fusarium, Pestalotiopsis and Russula published in 2020-2024. Full references and species included are listed in Suppl. material 1. Categories and scoring is explain in the Materials and methods part of the main text. https://binary.pensoft.net/file/1695005This dataset is made available under the Open Database License (http://opendatacommons.org/licenses/odbl/1.0/). The Open Database License (ODbL) is a license agreement intended to allow users to freely share, modify, and use this Dataset while maintaining this same freedom for others, provided that the original source and author(s) are credited. Michaela Caboňová, Sajeewa S. N. Maharachchimbukura, Chance R. Noffsinger, Miroslav Caboň, Xiang-Lin Li, Annemieke Verbeken, Marc Stadler, Chayanard Phukhamsakda, Yasmina Marin-Felix, Laura Guzmán-Dávalos, Nalin N. Wijayawardene, Slavomír Adamčík, Kevin D. Hyde
